# Case of the Bite of a Poisonous Snake Successfully Treated

**Published:** 1813-02

**Authors:** John Macrae

**Affiliations:** Civil Surgeon, Chittagong


*For the Medical and Physical Journal.*

### CASE *of the* BITE *of a* POISONOUS SNAKE *successfully treated.*

*By* JOHN MACRAE, *Esq. Civil Surgeon, Chittagong.*

THE disease in the human body, consequent to the bite of a serpent, from being so very rapid in its progress, has seldom afforded, to medical men, an opportunity of observing, and accurately ascertaining, its symptoms, in its more early stage; and, for that reason, a complete medical description of it is, as yet, a desideratum in physic.

It has been lately my lot to have the opportunity, in my own person, of ascertaining, from my immediate feelings, the several symptoms of this disorder, in its different stages, from the moment of receiving the poison into the habit, until (when it had nearly overcome the powers of life) it was happily counteracted by the use of medicine; and, my mind having been perfectly collected, (though so deeply interested in the result,) while I made my observations, they may be relied on as correct.

On the night of the 12th of May, on stepping into the southern verandah of my house, I observed a small snake, of a dark color, running along the terrace; and which, after several unsuccessful attempts, I at length hit and killed, with a small cane I had in my hand. Immediately thereafter, as I walked, I felt a slight uneasiness below the inner ankle of my right leg, as if I had taken off a bit of the skin, and this, I imagined, I had done, with the heel of the other foot, in my eagerness to kill the snake; and, therefore, after gently rubbing the part with my hand, I thought no more of it; but, in a few minutes, returned into the house, and began to undress to go to bed.

While undressing, I looked at my ankle, and could perceive a small red spot, where I felt the uneasiness, (pain I could not well call it,) but there was not the least appear-

1 ance ance of blood, nor was there any of the skin rubbed off. At this time I felt a great glow over my body, with a strong palpitation of the heart; but, as the night was warm and calm, I ascribed it to that cause, and the exertion I had made in killing the snake; and, under that impression, I went to bed. I could not, however, sleep; for the heat, and palpitation of the heart, soon increased so much, as to render me very restless; and I felt, besides, a very singular sensation, as if a warm fluid was circulating in my veins, to the very extremities of my fingers. This symptom, and the strong palpitation of the heart, which had become extremely disagreeable, were such as I had never experienced before; and, being of so very extraordinary a nature, I began to consider what could be the cause of them. I examined the state of my pulse, and found it to be full and strong, but indicating no disposition to fever. I then began to think if it was possible that the snake could have bitten me, without my being sensible of it at the moment, and that the symptoms I felt could be the effect of such an accident. The uneasiness at my ankle still continued as before, without appearing to increase; and I recollected, that, while endeavoring to kill the snake, it had once made a dart towards me, and got between my feet; but, as I did not perceive it to touch me, I was unwilling to ascribe, to so alarming a cause, the unusual sensations I felt; yet I could not otherwise account for them. While this reasoning passed rapidly in my mind, I was seized with a violent fit of vomiting, which at once solved all my doubts, as to the nature of my case; for, having observed sickness at stomach invariably to follow the bite of a snake, in all such patients as I had had the opportunity of seeing laboring under the effects of the poison, I was no longer at a loss to determine the cause of my disorder, and I accordingly got out of bed immediately to apply some remedy.

The first thing I did was to drink a strong mixture of brandy and water, with the view of relieving the sickness, which greatly oppressed me. At the same time, I walked briskly backwards and forwards in my room, in order to keep off the stupor, to which I knew there was so strong a tendency in this disorder. But a second fit of vomiting, more violent than the first, speedily came on; which entirely cleared the stomach of its contents, and left me in so very languid and exhausted a state, that, unable any longer to walk, I was obliged to throw myself upon a couch, and there to remain. The palpitation of the heart had now subsided, and was succeeded by a most distressing oppression in breathing, that compelled me to make frequent deep inspirations. The heat of my body had also abated, and was followed by a deadly coldness of the skin, and profuse perspiration, with a slow weak pulse; yet still I was sensible, in some degree, of the extraordinary feel as if a warm fluid was circulating in my veins, though I was becoming less so every moment.

Having a small medicine chest, fortunately, in the room where I lay, I directed a tea-spoonful to be given me of the *Spiritus Ammoniæ compositus*, in a Madeira glass full of water. This I took, in preference to the plain volatile alkali, from the idea, that the aromatic oil would render it more grateful to the stomach, which was still much oppressed with sickness. Finding that the first dose agreed with me, in about five minutes, (I imagine,) I took a second, and so on, a third, fourth, fifth, and sixth; when the medicine began to have a favorable effect. The first benefit I was sensible of deriving from it, was a relief from the sickness at the stomach; my breathing next became easier, my skin then began to recover its natural warmth, and the perspiration, with which I had been in a manner drenched, dried up by degrees. I still went on with the medicine, but at longer intervals, for every now and then I had a slight return of the oppression in breathing, which was immediately relieved on taking the alkali. I had thus gone on, until I had taken thirteen spoonfuls, or a wine glassful of the medicine, before I considered myself as out of danger; and, in proportion as I recovered, I became more and more sensible of the nauseous taste of the alkali, which latterly seemed to burn my throat as I swallowed it, though I could scarcely perceive the taste of the first dose I took, so totally gone was the nervous sensibility of my palate.

In the course of three hours from my receiving the bite, I was out of danger, but five hours had elapsed before I had entirely got the better of the effects of it. While lying on my couch, during the first three hours, I had my watch on the table before me, most anxiously looking forward to the passing time; for I thought, if the poison did not overpower me within that period, that I would have every chance of recovery, from the continued use of the medicine. I was very uneasy, lest I should lose my recollection, before I had taken the medicine in sufficient quantity to counteract the poison; as those around me, in that case, from not knowing my disorder, would most probably discontinue giving it to me; yet, from an extreme unwillingness to distress my family, by a disclosure of the nature of my illness, which happily had been considered, hitherto, a mere bilious attack, I never hinted to any one the true cause of it; nor would I send for any any of my friends in the place to attend me, as that would be indicating an apprehension of danger, which might prove equally alarming. For this reason, therefore, as well as from the certainty with which I was enabled to judge, by my immediate feelings, of the effect of the alkali, I took it in much larger and more frequent doses than I would have ventured to have prescribed for any other person in a similar situation; and to this circumstance of taking it in so unusually large a quantity, in so short a time, I have, under Providence, to ascribe my recovery; for, after the second fit of vomiting, I was sinking so fast, that nothing but so powerful a stimulus could have saved me.

The poison must have been of the most virulent nature, otherwise the very minute portion that could have been introduced into my habit would not have produced so immediate and violent an effect; for, on examining the part where I received the injury on the following morning, no appearance whatever of a wound was visible, but on touching the spot with the finger, and passing it gently along, a small rising like a pimple was perceptible, around which, on a close and minute inspection, a slight discoloration, of a livid appearance, was discernible. One fang only, and but the very point of that, could have wounded me; for the snake being small, and the skin below my ankle in a state of great tension, as I stood, the animal was unable to lay hold of me; but, in the attempt to do so, it struck against my leg with the point of this fang, and that so slightly as to draw no blood; and therefore I did not feel it at the moment, nor was I aware of it afterwards, when I looked at my ankle, while undressing to go to bed.

Had a larger quantity of the poison entered my habit, there can be no doubt but that it would have proved fatal, before I could have had any suspicion of danger, or have applied a remedy. I have not ascertained the species of the snake, having thrown it away, without examination, immediately as I killed it; but a bearer, who was with me, and saw it, calls it *Chopperpoora*, and says it is peculiar to *Choppers* of old buildings. The outer verandah of my house is covered with grass, from which it most likely came to the terrace.

I continued, for several days after the accident, in a state of the greatest lassitude, but felt no other unpleasant symptom, and this gradually wore off, until I recovered my usual health, without the aid of any medicine.

From the foregoing statement it appears, that the first effect of the poison, on being received into the body, was to excite the action of the heart and arteries, and to produce a

 great great heat over the whole body; and, as a similarity of effect proves a similarity of cause, and the effect of all stimuli is to excite, it follows that the poison of the serpent is a stimulus, and of the most powerful nature, that destroys life by its excess.

The symptoms of debility which so immediately ensued, viz. the sickness, profuse cold sweat, and low pulse, are also consequent to the application in excess of other stimuli; and, according to the greater or less degree of this excess, so is the state of debility that ensues, and death follows, sooner or later, from it. There are instances recorded where the poison of the serpent proved so quickly fatal, as to preclude the possibility of applying any remedy; but, in general, some hours elapse, from the time of receiving it into the habit, before it destroys life, and there is, consequently, an opportunity afforded of counteracting its effects, when assistance is at hand. The volatile alkali has been long in use in such cases, and has been frequently administered with the greatest success, but, unfortunately, our knowledge of the disorder consequent to the bite of the snake has been so imperfect, and the principle upon which its cure had been accomplished (whenever this happened) had been so little understood, as to have produced much indecision in our practice; and this valuable medicine, therefore, has been on many occasions either entirely laid aside, or it has been given in such trifling doses as could do no good, and it has, in consequence, been considered as of very doubtful virtue, if possessing any. Indeed, this want of confidence prevails with respect to the efficacy of every description of medicine, in the cure of this alarming disease. But, in the foregoing case, is given a connected detail of symptoms, as they succeeded each other, from the earliest stage, with an accurate account of the operation of the alkali from its first perceptible effect in counteracting them; and, having thus a complete history, as well of the disorder as of its remedy, we are thereby enabled to form a correct opinion of both, and to act accordingly upon a plan of cure, equally fixed and systematic, in this as in any other disease incident to the human body, a circumstance, it is to be hoped, that cannot but prove of much future benefit to mankind.

In prescribing and administering medicine for this disorder, much decision and promptness are necessary, because its progress is so very rapid. This is a point that cannot be too strongly impressed upon the mind. Our remedy must be powerfully applied, before the vital powers are so far gone as to become insensible of its effect. For this reason

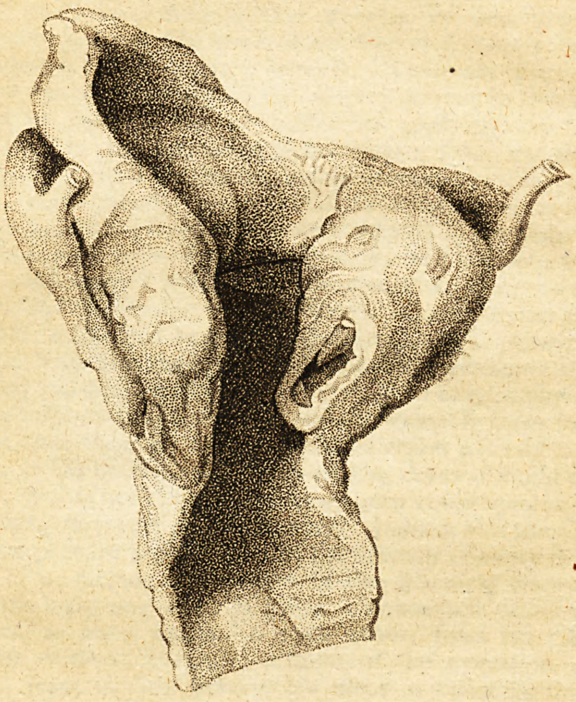

*Cooper, Sculp$^{t}$*

*Published 1813, by J. Souter, Paternoster Row.*

such stimuli as are of most immediate operation are to be preferred, and the volatile alkali, on that account, is so particularly useful, as (no doubt) æther will be found to be. But, whatever medicine is administered, it is to be given in large and frequently-repeated doses, until we perceive that a favorable change is produced. The state of the skin, and of the pulse, with the patient's remarks as to his feelings, are to be our guide, and to direct our judgment in this; for, until a return of the natural warmth of the skin, and an increase in the strength and quickness of the pulse take place, we ought to push our remedies. And, so far from considering the sickness as the consequence of giving medicine, and therefore an objection to our further doing so, it is the very reason why we should continue a more powerful application of it; because, the sickness being the effect of the debility induced by the poison, the continuance of it proves that a sufficient stimulus has not been applied to overcome this debility, and therefore more is necessary to produce that end. In short, the stimulus must be proportioned to the degree of debility to be overcome; and on the judgment with which this is done, depends entirely our success in the cure.*

* This case was addressed, by Mr. Macrae, to John Fleming, esq. President of the Medical Board, Fort William, July 22d, 1809.

